# The Influence of Counterfactual Comparison on Fairness in Gain-Loss Contexts

**DOI:** 10.3389/fpsyg.2017.00683

**Published:** 2017-05-09

**Authors:** Qi Li, Chunsheng Wang, Jamie Taxer, Zhong Yang, Ya Zheng, Xun Liu

**Affiliations:** ^1^Key Laboratory of Behavioral Science, Institute of Psychology, Chinese Academy of Sciences (CAS)Beijing, China; ^2^Department of Psychology, University of Chinese Academy of Sciences (UCAS)Beijing, China; ^3^Stanford Psychophysiology Laboratory, Department of Psychology, Stanford UniversityStanford, CA, USA; ^4^Department of Psychology, Dalian Medical UniversityDalian, China

**Keywords:** counterfactual comparison, social decision-making, ultimatum game, loss context, gain context

## Abstract

Fairness perceptions may be affected by counterfactual comparisons. Although certain studies using a two-player ultimatum game (UG) have shown that comparison with the proposers influences the responders' fairness perceptions in a gain context, the effect of counterfactual comparison in a UG with multiple responders or proposers remains unclear, especially in a loss context. To resolve these issues, this study used a modified three-player UG with multiple responders in Experiment 1 and multiple proposers in Experiment 2 to examine the influence of counterfactual comparison on fairness-related decision-making in gain and loss contexts. The two experiments consistently showed that regardless of the gain or loss context, the level of inequality of the offer and counterfactual comparison influenced acceptance rates (ARs), response times (RTs), and fairness ratings (FRs). If the offers that were received were better than the counterfactual offers, unequal offers were more likely to be accepted than equal offers, and participants were more likely to report higher FRs and to make decisions more quickly. In contrast, when the offers they received were worse than the counterfactual offers, participants were more likely to reject unequal offers than equal offers, reported lower FRs, and made decisions more slowly. These results demonstrate that responders' fairness perceptions are influenced by not only comparisons of the absolute amount of money that they would receive but also specific counterfactuals from other proposers or responders. These findings improve our understanding of fairness perceptions.

## Introduction

Individuals often reject an unequal distribution even at a cost to themselves. This phenomenon has been examined using the ultimatum game (UG) (Sanfey et al., 2003; Tabibnia et al., 2008; Dulebohn et al., 2009; Güroglu et al., 2010, 2011). In this game, two players divide a sum of money. The first player, the proposer, proposes how the money should be divided. The second player, the responder, accepts or rejects the proposed division. If the responder accepts, the money is divided as proposed. If the responder rejects, both players receive nothing. To the responder, fairness means an equal distribution between the proposer and the responder (Alexopoulos et al., 2012; Zheng et al., 2015). Standard economic models predict that the responder makes the rational decision to maximize his/her benefits and that any monetary amount is preferable to none. However, multiple studies that used the UG have found that many responders reject unequal offers, especially offers below 20% of the total (Güth et al., 1982; Thaler, 1988; Camerer and Thaler, 1995); this finding reflects the important role of perceived fairness when making such decisions.

Fairness perceptions are driven by comparing an interaction's actual benefit with the benefit that could have been obtained (counterfactual comparison) (Sandbu, 2007; Nicklin et al., 2011). In some studies that use a modified, two-person UG, the proposer selects one of two paired offers. For example, an unfair offer of seven coins for the proposer and three coins for the responder (7:3) is paired with an alternative: either a fair alternative (5:5) or a highly unfair alternative (9:1). These studies have demonstrated significant counterfactual comparison effects, finding that responders are more likely to accept an unfair offer when the alternative is highly unfair than when it is fair (Falk et al., 2003; Radke et al., 2012). In the two-person UG, the counterfactual comparison effect comes from speculating the proposer's intention, whereas in the three-person UG, this effect comes from the comparison between the responder's offer and the third player's offer (Wright et al., 2011; Du et al., 2013). Studies have discovered that regardless of whether the UG includes a third player as another responder (Knez and Camerer, 1995; Kagel and Wolfe, 2001), a group of proposers (Wright et al., 2011; Du et al., 2013), or an average amount (Wu et al., 2011), the responder is more likely to reject the offer if there is a better alternative offer from a third player. However, worse alternative offers to another powerless responder are ignored (McDonald et al., 2013). Only when responders are confronted with inequality do they care about unequal alternative offers from another powerless responder and demonstrate increased rejection behavior (Alexopoulos et al., 2012, 2013). Although previous studies have investigated whether and how a responder's behavioral response to unfairness is affected by a third player's offer, the third player is a powerless responder in these studies (Alexopoulos et al., 2012, 2013; McDonald et al., 2013) or an abstract average amount is involved (Wu et al., 2011). To our knowledge, a third player as a powerful responder or proposer has received little attention in the thousands of experimental studies that have used UG.

Although the influence of fairness on decisions has been examined extensively in gain contexts (Güth et al., 1982; Camerer, 2003; Sanfey et al., 2003), little attention has been paid to the influence of fairness on decisions in loss contexts. According to prospect theory, losses loom larger than gains, and people prefer to avoid losses rather than to acquire equivalent gains (Güth et al., 1982; Buchan et al., 2005; Wu et al., 2011). In a two-person UG, money is divided between the proposer and the responder. In this situation, for the responder, fairness is the one of the important norms to direct his/her judgment (Fischbacher et al., 2013; Hertwig et al., 2013). In comparison with gain contexts, loss contexts lead individuals to perceive stronger unfairness and to reject more unequal offers (Guo et al., 2013; Sarlo et al., 2013; Tomasino et al., 2013). In contrast to a two-person UG, in a three-person UG, responders hope to obtain higher payoffs, and they hope that their divisions and earnings are equal to or better than the divisions and earnings of the third player (Shupp et al., 2006; Alexopoulos et al., 2012, 2013; McDonald et al., 2013; Wang et al., 2014). As responders, they cannot control the divisions by themselves; they must accept the offers to increase benefits or reduce debts. Therefore, we infer that responders may be less likely to reject offers in a loss context than in a gain context to avoid widening the gap in earnings with a third player due to a rejected offer. To the best of our knowledge, this study is the first to explore the influence of counterfactual comparison on fairness perceptions using a three-person UG in a loss context.

Acceptance rates (ARs) and fairness ratings (FRs) are widely used indices of fairness perception (Moretti and Di Pellegrino, 2010). Moreover, response times (RTs) have regularly been employed as a measure in its own right to explain the underlying process of fairness perception and to test fairness models in investigations of fairness in decision-making (van 'tWout et al., 2005; Wright et al., 2011). People's deliberations may be influenced by various criteria, and the examination of more criteria is associated with requiring longer times to make a decision (Fischbacher et al., 2013; Hertwig et al., 2013). In order to obtain a comprehensive understanding of the influence of counterfactual comparisons on fairness perceptions, we decided to complement the widely used measures ARs and FRs by the RTs participants needed when making their acceptance decisions.

As responders in UGs, people are often understood as using their own internal fairness yardstick to judge the acceptability of offers. Therefore, our research aimed to investigate whether counterfactual comparisons in gain and loss contexts could affect people's FRs (and subsequent decisions) in a modified three-person UG in which the third player was either another responder (Experiment 1) or another proposer (Experiment 2). In contrast to previous three-person UG studies (Güth et al., 2007), a three-person UG with multiple responders is a game in which there is one proposer who proposes separate offers to two responders. The two responders independently decide whether to accept or reject the offers. A three-person UG with multiple proposers is a game in which there are two proposers who propose two divisions and present them to one responder simultaneously. The computer selects one of the offers for the responder, who decides whether to accept the selected offer. Given these considerations, we examined the following three research questions. First, do counterfactual offers influence a responder's willingness to accept equal or unequal offers (i.e., compared with seeing other worse/better offers)? Second, does a counterfactual offer affect not only the willingness to accept or reject but also the decision processes (i.e., FRs and RTs)? Third, are the effects of counterfactuals robust across different contexts (i.e., gain/loss contexts and whether there are multiple responders/proposers)?

## Materials and methods

### Participants

Non-psychology and economic majors at a university in Beijing were recruited as participants through the university's Bulletin Board System. None of the participants reported that they had previously been part of similar experiments. Overall, 58 (21 males, M ± SE = 22.5 ± 0.31 years) and 67 (25 males, M ± SE = 22.4 ± 0.27 years) university students were included in Experiment 1 (a UG with multiple responders) and Experiment 2 (a UG with multiple proposers), respectively. All participants were right-handed and had normal or corrected-to-normal vision. They had no history of neurological or psychiatric disorders. The participants voluntarily enrolled in the study and signed a written consent form. This study was conducted in accordance with the Declaration of Helsinki, and the procedures were approved by the local institutional review board.

### Attention and comprehension checks

#### Attention check

To ensure that the participants paid attention over the course of the relatively large number of trials and to enhance the comparison between the two offers, for each trial, the participants were asked to judge the offer size. Specifically, the participants had to judge which offer would allow them to gain more in the gain context (or lose less in the loss context). The participants were asked to press the F key with their left index finger if the left offer was more (or less) and to press the J key with their right index finger if the right offer was more (or less). Similarly, half of the participants needed to judge which offer would allow them to gain less in the gain context (or lose more in the loss context). In Experiments 1 and 2, the accuracies of the offer size judgments (M ± SE) were 97 ± 0.3% and 97 ± 0.4%, respectively. One participant in Experiment 1 and two participants in Experiment 2 were excluded from further analyses because the mean accuracies of their judgments of offer size were less than 80%.

#### Comprehension check

To ensure that all participants fully understood the task, a practice block of three trials that was identical to the experimental blocks was administered after the participants read the instructions. The participants were told that the purpose of the practice trials was to help them to become familiar with the tasks. In contrast to the experimental trials in which the offers were given by the proposers, in the practice trials, the offers were randomly generated by the computer. After the practice trials, the researchers answered any questions regarding the experiment and ensured that all participants completely understood the rules; only after this process did the experimental blocks begin.

### Procedure

Before participating in the study, the participants were asked to describe themselves in a self-introduction, including their major, upbringing, education, personality, and hobbies. The participants were informed that their self-introduction would be anonymously presented to all proposers and that the proposers would decide how to divide the money with them according to the information in their self-introduction. When they arrived at the experiment, the participants were told that we had given their self-introductions along with those of the other participants (Experiment 1) or their self-introductions (Experiment 2) to more than one hundred proposers to allow the proposers to decide how to distribute the money in advance. Furthermore, the participants were told that these offers from the different proposers had been entered into a computer for display. The participants were also told that the proposers were given money according to the responders' decisions by Alipay at the end of both experiments. Therefore, the participants knew that all offers were determined ahead of time and that there were not hundreds of people who were simultaneously participating in the experiments. After the participants finished the experiment, they were informed that there were no real proposers and that all of the participants had been given the same offers regardless of the content of their self-introductions.

**Figure 1:**
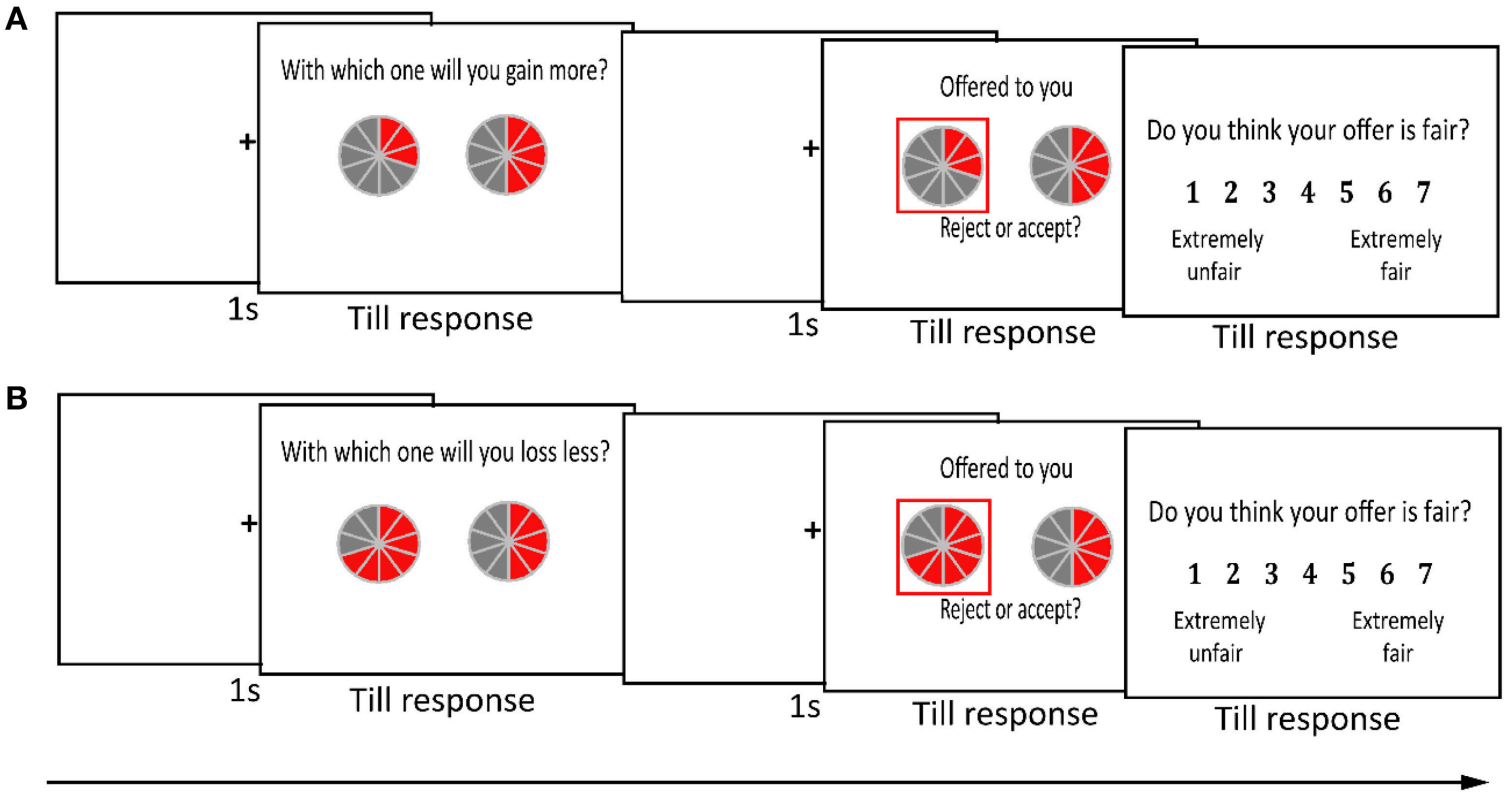
**Experimental procedure for both experiments**. The participants played an ultimatum game in a **(A)** gain context or **(B)** loss context. Two pies represented two offers from one proposer who proposed to two responders in Experiment 1, and two pies represented two offers from two different proposers in Experiment 2. The offer to the participant was framed in a red square and could be accepted or rejected. Each pie was divided into 10 equal slices and represented ¥10 in total. The participant's slices were in red, and the proposer's slices were in gray.

On the day of the experiment, the rules of the UG game were explained to the participants (Figure 1). Specifically, each participant in Experiment 1 was informed that in each trial, the proposer would present two offers. The total amount of each offer was ¥10. Two pies were used to represent the two offers. One pie was enclosed in a red square and displayed the offered split between the proposer and the participant (the responder), and the other pie presented the offered split between the proposer and another responder (a pseudo-subject). Each pie was divided into 10 equal slices, with each slice representing ¥1. Gray slices and red slices represented the payoffs to a proposer and a responder, respectively. In the gain context, accepting the offer led to the suggested division of the gain, whereas rejection resulted in the proposer and responder each receiving nothing. In the loss context, accepting the offer led to the suggested division of the loss, whereas rejection resulted in the proposer and responder each incurring the entire loss. To ensure that the participants would independently compare their own payoffs and not infer a norm from other peoples' behavior, they were told that the decisions of other responders would not affect their payoff and that proposers and other responders would not know the participants' decisions and vice versa. Furthermore, to avoid an influence of the previous trial on the current trial, the participants were asked to treat each trial as if it were the only trial that would determine how much money they and their proposer would receive at the end of the experiment. These manipulations have commonly been used in research with UGs (Wright et al., 2011; Feng et al., 2015; Ma et al., 2015; Ma and Hu, 2015). In addition, to increase the strength of any counterfactual comparison effect, two participants took part in the experiment at the same time and were told that they were of similar age, educational background, and university ranking. Before the formal experiment, the participants were told that they would be given a basic payment for their participation (¥40 RMB), plus or minus the amount of money that was gained or lost from a random selection of 10% of the formal experimental trials.

Both experiments consisted of 160 trials that were divided evenly into four runs, half with a gain context and half with a loss context, as well as 20 buffers. For all payoff combinations, the first number represented the payoff for the proposer, and the second number represented the payoff for the responder. In the gain context, when the responder's offer was equal (+5:+5), the counterfactual offer was one of the following conditions in comparison: moderately disadvantageous (+7:+3); slightly disadvantageous (+6:+4); slightly advantageous (+4:+6); or moderately advantageous (+3:+7). When the responder's offer was unequal (+7:+3), the counterfactual offer was one of the following conditions in comparison: moderately disadvantageous (+9:+1); slightly disadvantageous (+8:+2); slightly advantageous (+6:+4); or moderately advantageous (+5:+5). In the loss context, when the responder's offer was equal (−5:−5), the counterfactual offer was one of the following conditions in comparison: moderately disadvantageous (−3:−7); slightly disadvantageous (−4:−6); slightly advantageous (−6:−4); or moderately advantageous (−7:−3). When the responder's offer was unequal (−3:−7), the counterfactual offer was one of the following conditions in comparison: moderately disadvantageous (−1:−9); slightly disadvantageous (−2:−8); slightly advantageous (−5:−5); or moderately advantageous (−4:−6). There were 10 trials for each condition. The context order was counterbalanced among the participants, and different types of offers were mixed in pseudorandom order, with the restriction that no more than three consecutive trials had the same offer.

For each trial, a fixation cross was presented for 1.5 s. Then, two offers were presented on the screen. After the attention check for the judgment of offer size was made, the responder's offer was framed by a white square. Following a fixation cross for 1 s, the responder's offer for the participants for which the location was counterbalanced was presented with a red square. The participants were required to press the F key with their left index finger to accept the offer and to press the J key with their right index finger to reject the offer. The response buttons were counterbalanced among the participants. At the end of each trial, the final outcome was shown until the participant reported the fairness rating of the responder's offer on a 7-point scale that ranged from 1 (extremely unfair) to 7 (extremely fair). After the experiment, the participants were debriefed. All of them believed that they played the game with real proposers and another responder.

For Experiment 2, the procedure was similar to Experiment 1. The only difference was that each of two proposers provided one offer to the participant. In each trial, two pies represented separate offers from two proposers, and one of the offers was randomly selected (i.e., enclosed in a red square) by the computer for the participant.

## Results

The mean ARs, RTs (log RTs; transformed using a logarithmic function), and FRs of each condition are shown in Table 1 (Experiment 1) and Table 2 (Experiment 2). To exclude the contribution of the attention check, we conducted two independent samples *T*-tests to test the influences of judgments of offer size and response buttons on the ARs (and RTs and FRs). We did not find any significant difference (*ps* > 0.05). Then, a series of 2 (responder's offer: equal vs. unequal) × 4 (counterfactual offer: moderately advantageous vs. slightly advantageous vs. slightly disadvantageous vs. moderately disadvantageous) × 2 (context: gain vs. loss) repeated-measure ANOVAs were conducted on the ARs, RTs, and FRs. A two-step cluster analysis was conducted to classify specific response strategies into different groups based on Schwarz's Bayesian information criterion (BIC) and the highest log-likelihood distance measures (ratio of distance measures, RDM) by pooling together the ARs of the different conditions in both experiments. As an additional between-group factor, a mixed ANOVA was further conducted to delineate the differences in the ARs among the different strategy groups. In addition, Pearson correlation coefficients of the ARs, RTs, and FRs were obtained by pooling together all data. Statistical significance was defined at the 0.05 level (two-tailed test). The analysis of the participants' behavioral changes over time is shown in the Supplementary materials.

**Table 1:** **Mean (standard errors) ARs, RTs, and FRs of all conditions in Experiment 1**.

**Responder's offer**	**Counterfactual offer**	**ARs**	**RTs**	**FRs**
+5:+5	+7:+3	0.97 (0.01)	3.01 (0.02)	4.84 (0.09)
	+6:+4	0.98 (0.01)	3.03 (0.02)	4.72 (0.10)
	+4:+6	0.97 (0.01)	3.01 (0.02)	4.28 (0.09)
	+3:+7	0.96 (0.01)	3.05 (0.02)	4.26 (0.11)
+7:+3	+9:+1	0.87 (0.03)	3.08 (0.03)	3.66 (0.13)
	+8:+2	0.86 (0.03)	3.10 (0.03)	3.64 (0.13)
	+6:+4	0.78 (0.04)	3.11 (0.03)	3.21 (0.10)
	+5:+5	0.71 (0.05)	3.15 (0.02)	3.05 (0.10)
−5:−5	−3:−7	0.97 (0.01)	3.09 (0.02)	4.17 (0.10)
	−4:−6	0.99 (0.01)	3.07 (0.02)	4.11 (0.09)
	−6:−4	0.99 (0.00)	3.13 (0.02)	3.75 (0.10)
	−7:−3	0.98 (0.01)	3.13 (0.02)	3.67 (0.10)
−3:−7	−1:−9	0.92 (0.02)	3.14 (0.02)	2.97 (0.12)
	−2:−8	0.91 (0.02)	3.15 (0.02)	2.92 (0.11)
	−4:−6	0.88 (0.03)	3.22 (0.02)	2.69 (0.11)
	−5:−5	0.81 (0.04)	3.23 (0.02)	2.60 (0.11)

**Table 2:** **Mean (standard errors) ARs, RTs, and FRs of all conditions in Experiment 2**.

**Responder's offer**	**Counterfactual offer**	**ARs**	**RTs**	**FRs**
+5:+5	+7:+3	0.97 (0.01)	2.98 (0.03)	4.93 (0.1)
	+6:+4	0.98 (0.01)	2.94 (0.03)	4.88 (0.10)
	+4:+6	0.99 (0.00)	2.97 (0.02)	4.45 (0.12)
	+3:+7	0.96 (0.01)	2.98 (0.02)	4.27 (0.13)
+7:+3	+9:+1	0.90 (0.03)	3.00 (0.03)	3.53 (0.12)
	+8:+2	0.89 (0.03)	3.01 (0.03)	3.47 (0.11)
	+6:+4	0.86 (0.03)	3.03 (0.03)	3.14 (0.09)
	+5:+5	0.79 (0.04)	3.04 (0.03)	2.95 (0.11)
−5:−5	−3:−7	0.99 (0.00)	3.01 (0.02)	4.45 (0.11)
	−4:−6	1.00 (0.00)	2.99 (0.02)	4.38 (0.11)
	−6:−4	0.98 (0.01)	3.05 (0.02)	4.07 (0.11)
	−7:−3	0.97 (0.01)	3.06 (0.02)	3.89 (0.12)
−3:−7	−1:−9	0.84 (0.04)	3.06 (0.02)	3.17 (0.13)
	−2:−8	0.84 (0.04)	3.04 (0.02)	3.03 (0.13)
	−4:−6	0.84 (0.04)	3.13 (0.02)	2.66 (0.10)
	−5:−5	0.80 (0.04)	3.15 (0.02)	2.58 (0.11)

### Responders' ARs influenced by the counterfactual offers

**Figure 2:**
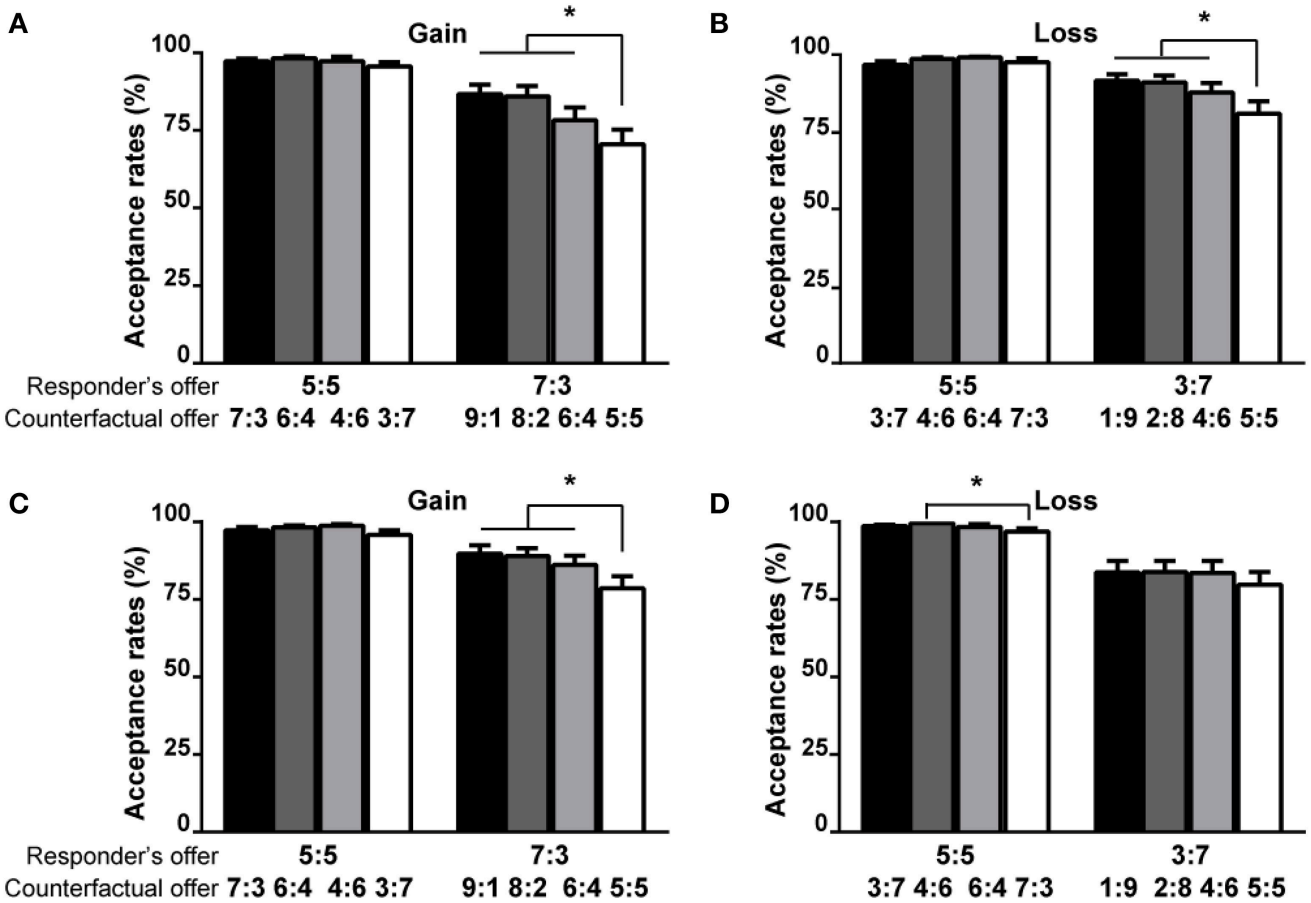
**The influence of counterfactual offers on the ARs of the responder's offer for Experiment 1 (A,B) and Experiment 2 (C,D)**. The first number of each vector represents the payoff for the proposer, and the second number of each vector represents the payoff for the responder. The error bars from the ANOVAs represent the standard errors of the means. The asterisk (^*^) represents the significant difference of the *post-hoc* Bonferroni tests at the *p* < 0.05 level.

**Figure 3:**
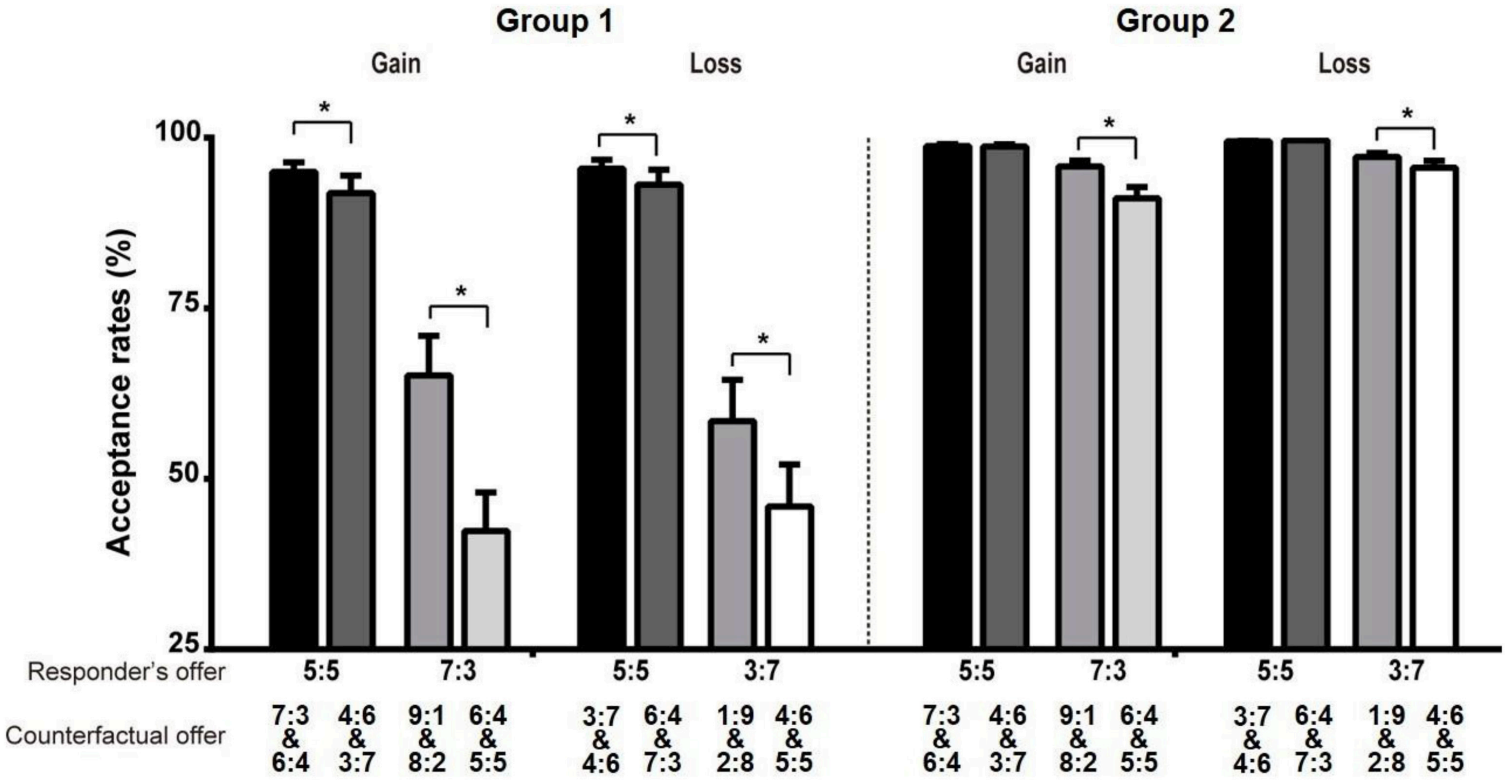
**The influence of counterfactual offers on the ARs of the responder's offer in distinct strategy groups**. The two-step cluster analysis yielded two groups (BIC = 554.47, RDM = 3.93). Group 1 contained 32 (25.6%) participants, and Group 2 contained 93 (74.4%) participants. The first number of each vector represents the payoff for the proposer, and the second number of each vector represents the payoff for the responder. The error bars from the ANOVAs represent the standard errors of the means. The asterisk (^*^) represents the significant difference of the *post-hoc* Bonferroni tests at the *p* < 0.05 level.

The results from multiple responders revealed that for the equal offers, there was no significant difference in AR among the distinct counterfactual offers, whereas for the unequal offers, the responders' ARs were significantly lower in the moderately advantageous condition than in the three other conditions (Figures 2A,B). The results from multiple proposers showed that for equal offers, the AR in the moderately advantageous condition was significantly lower than that in the slightly disadvantageous condition in the loss context (Figure 2D), whereas for unequal offers, the AR in the moderately advantageous condition was significantly lower than those in the other three conditions in the gain context (Figure 2C). Furthermore, for Group 1, the ARs in the advantageous conditions were significantly lower than those in the disadvantageous conditions (Figure 3). For Group 2, the ARs in the advantageous conditions were significantly lower than those in the disadvantageous conditions only for unequal offers (Figure 3, Table 3).

**Table 3:** **Results of auto-clustering**.

**Number of clusters**	**Schwarz's bayesian criterion (BIC)**	**BIC change**	**Ratio of BIC changes**	**Ratio of distance measures**
1	766.392			
2	554.471	−211.921	1.000	3.925
3	558.044	3.573	−0.017	2.022
4	598.852	40.808	−0.193	1.104
5	643.096	44.244	−0.209	1.260
6	694.151	51.055	−0.241	1.345
7	751.931	57.780	−0.273	1.050
8	810.638	58.707	−0.277	1.306
9	873.695	63.057	−0.298	1.046
10	937.376	63.680	−0.300	1.121
11	1002.521	65.145	−0.307	1.017
12	1067.870	65.349	−0.308	1.116
13	1134.458	66.588	−0.314	1.085
14	1201.881	67.423	−0.318	1.089
15	1270.111	68.230	−0.322	1.258

### The decision processes of the log RTs and FRs affected by the counterfactual offers

**Figure 4:**
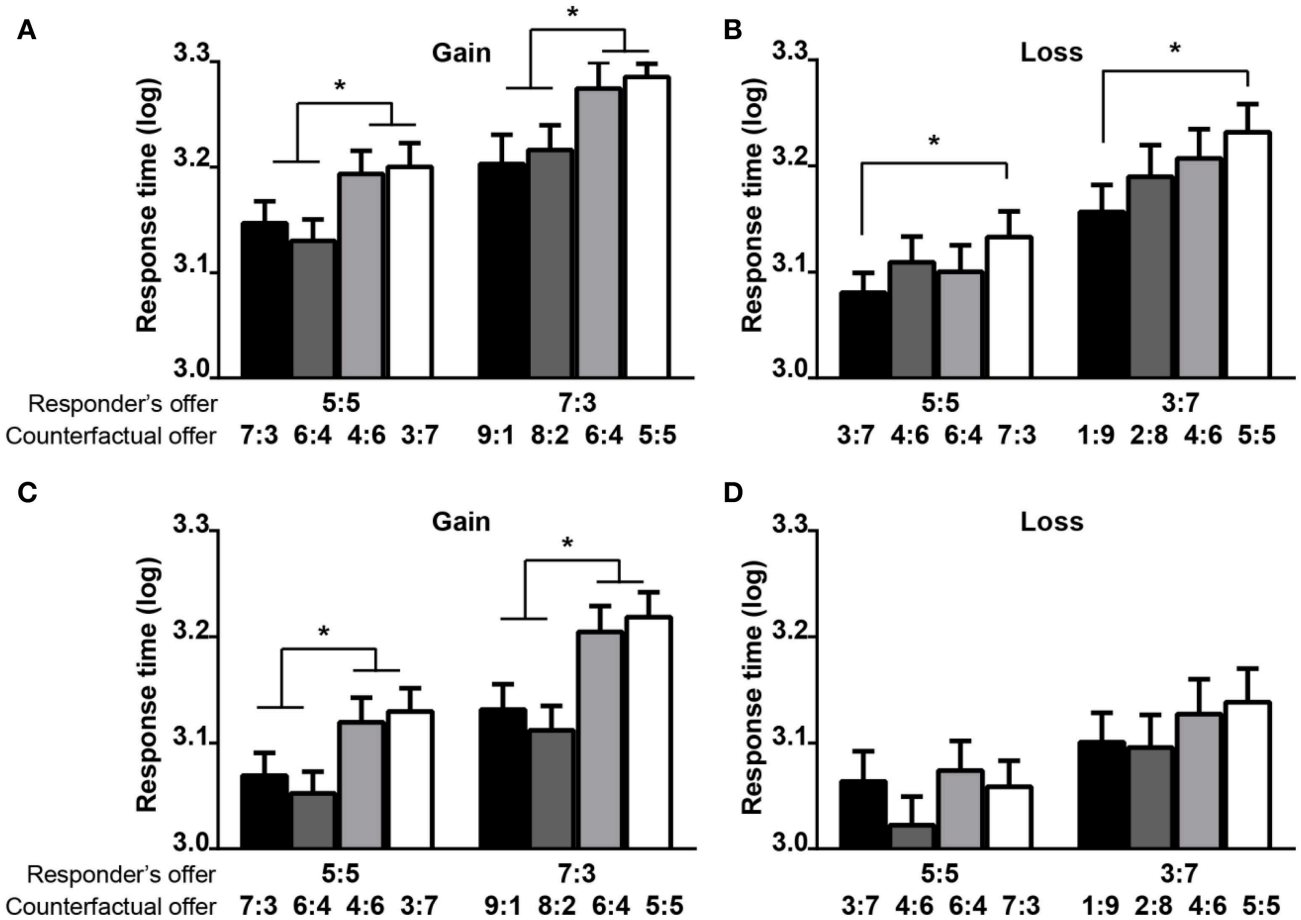
**The influence of counterfactual offers on the log RTs of the responder's offer in Experiment 1 (A,B)** and Experiment 2 **(C,D)**. The first number of each vector represents the payoff for the proposer, and the second number of each vector represents the payoff for the responder. The error bars from the ANOVAs represent the standard errors of the means. The asterisk (^*^) represents the significant difference of the *post-hoc* Bonferroni tests at the *p* < 0.05 level.

The results from multiple responders revealed that in the gain context, the RTs in the disadvantageous conditions were significantly shorter than those in the advantageous conditions (Figure 4A). In the loss context, the RTs in the moderately disadvantageous conditions were significantly shorter than those in the moderately advantageous conditions (Figure 4B). The results from multiple responders showed that in the gain context, the RTs in both disadvantageous conditions were significantly shorter than those in both advantageous conditions (Figure 4C). In the loss context, there was no significant difference of RTs among the different counterfactual offers (Figure 4D).

**Figure 5:**
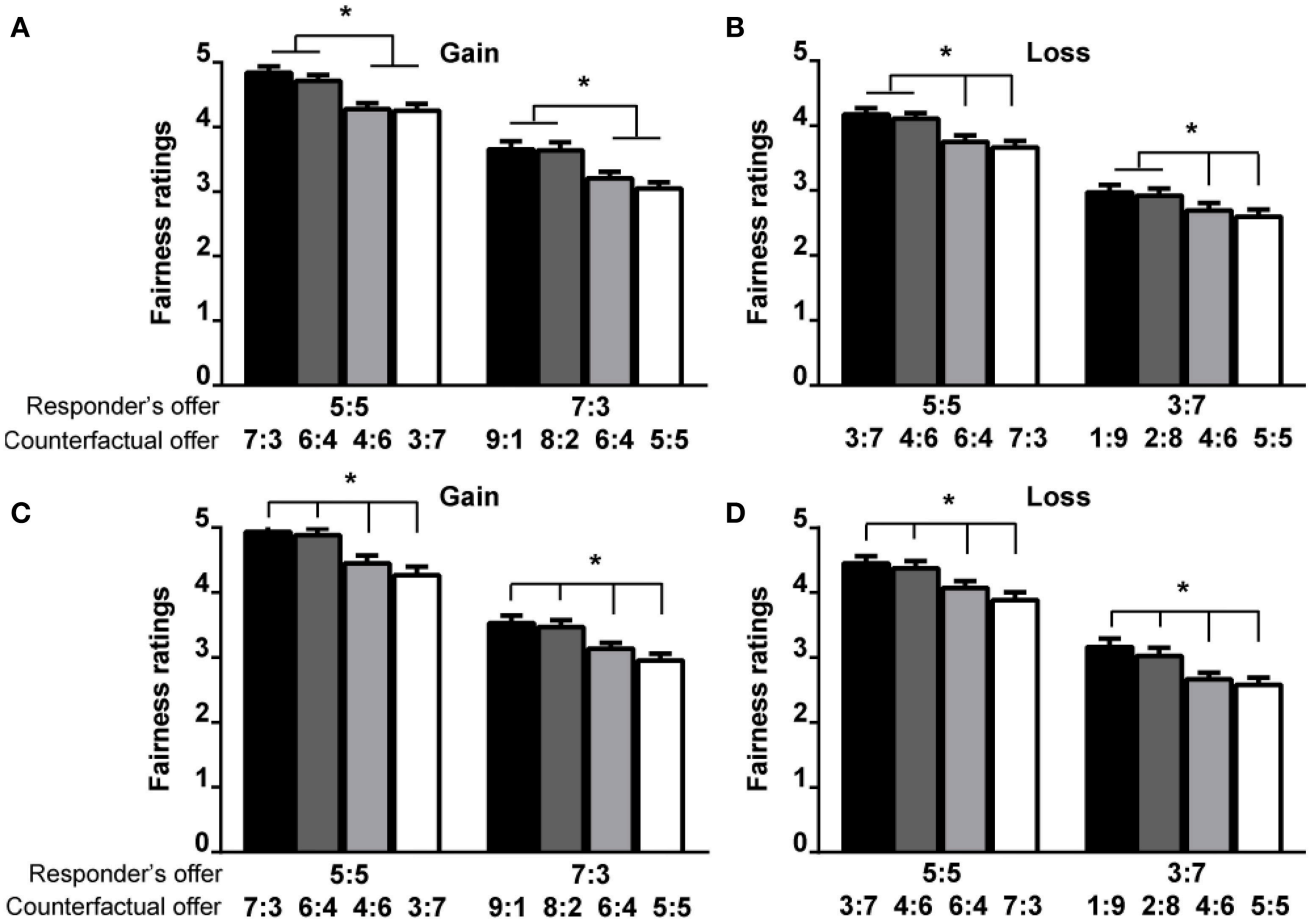
**The influence of counterfactual offers on the FRs of the responder's offer in Experiment 1 (A,B)** and Experiment 2 **(C,D)**. The first number of each vector represents the payoff for the proposer, and the second number of each vector represents the payoff for the responder. The error bars from the ANOVAs represent the standard errors of the means. The asterisk (^*^) represents the significant difference of the *post-hoc* Bonferroni tests at the *p* < 0.05 level.

The results from multiple responders revealed that in the gain context, the FRs in the two disadvantageous conditions were significantly higher than those in the two advantageous ratings (Figure 5A). In the loss context, the FRs of the moderately disadvantageous, slightly advantageous and moderately advantageous conditions decreased in turn, whereas there was no significant difference in FR between the two disadvantageous conditions (Figure 5B). The results from multiple proposers showed that the FRs in the moderately disadvantageous, slightly disadvantageous, slightly advantageous and moderately advantageous conditions decreased progressively (Figures 5C,D). Furthermore, for unequal offers, the ARs, RTs, and FRs were related to one another.

### The robust counterfactual effects in the context variables

The results of the ARs, RTs, and FRs consistently showed that the counterfactual effects were significant to the context variables, such as the gain/loss or multiple responders/proposers contexts (Figures 2–5).

## Discussion

This study used a modified three-person UG with multiple responders or proposers to investigate how counterfactual comparisons in gain and loss contexts affect individuals' perceptions of fairness and decision-making. Consistent with previous studies (van 'tWout et al., 2005; Polezzi et al., 2008; Wright et al., 2011), the results of the two experiments indicated that the levels of unequal offers influenced people's fairness evaluations. More importantly, we found that emphasizing the counterfactual offers modulated the responders' willingness to accept unequal offers. These effects of counterfactuals were generally robust to the context variables such as the gain/loss or multiple responders/proposers contexts. In addition, individual decision processes, such as the FRs and RTs, were modulated by the counterfactual offers. In sum, our results are consistent with the idea that people's fairness evaluations are influenced by the specific counterfactuals that they are faced with when making their evaluations instead of merely comparing the offer to any form of internalized fairness standard.

### Responders' ARs influenced by the counterfactual offers

Our research suggests that counterfactual comparisons may affect responders' willingness to accept offers from proposers. The results of the two experiments indicate that for unequal offers, individuals accept less in the gain and loss contexts when they know that the counterfactual offers to the third player contain more. Consistent with previous multiple-person UG studies in which the allocation of a third player with decision power (Knez and Camerer, 1995; Du et al., 2013; Zheng et al., 2015) or an average amount (Bohnet and Zeckhauser, 2004; Wu et al., 2011) were presented, our research showed that people were less likely to accept an unequal offer if it left them with a more inequitable payoff relative to their peers, and they demanded less when they knew that their peers were offered less regardless of the gain or loss contexts. However, previous studies have found that responders appeared to ignore a powerless responder when the latter received less than the responders received, and they cared about the powerless responder only when both of them were confronted with inequality, which caused an increase in rejection rates (Kagel and Wolfe, 2001; Alexopoulos et al., 2012, 2013; McDonald et al., 2013).

Why do our results differ from these studies even though the reverse results were found for the ARs when unequal offers were proposed to the responder and the third player? One possible reason is that in these three-person UG studies, the responder had veto power, whereas the third player was a powerless responder (non-responder) who could not make an independent decision. Therefore, the responder may not have regarded alternative offers from the powerless responder as a comparable reference agent. When both the responder and the third player received unequal offers, the responder regarded himself/herself as the representative of the group and the third player as one of the members of his/her group. In this situation, the responder may believe that he/she has more responsibility to maintain the fairness norm and may less frequently accept unequal offers. In our research, the responder and the third player had the same payoffs, and both of them could independently accept or refuse the proposed division. Therefore, the responder would want to be normatively entitled to earn as much as the other player in similar circumstances (Festinger, 1954; Bohnet and Zeckhauser, 2004). Compared with the counterfactual offers from the third player, unequal offers plus disadvantageous payoffs increased the responders' inequality aversion and thus promoted the rejection of unequal behavior. In contrast, unequal offers plus advantageous payoffs reduced the responders' inequality aversion and thus promoted the acceptance of unequal behavior.

In contrast to unequal offers, when individuals are faced with equal offers, their willingness to accept equally divided offers is less subject to the influence of counterfactual comparison. Considering that most of the participants would like to accept equal offers (Xiao and Houser, 2005; Dreher et al., 2016), we speculate that the ARs in equal offer conditions are very high and reach a ceiling level. The ceiling effect of the ARs from equal offers may cause the counterfactual offers to no longer have an effect and to no longer be measured or estimated.

Furthermore, we found that although different response strategies were used by the two groups, fairness perceptions in both groups were affected by counterfactual comparisons. From the results of the cluster analysis, we observed that the individuals in the two groups had a distinct and common internalized fairness standard. First, we observed some distinct behavioral patterns between the two groups. The individuals in Group 1 tended to reject more unequal offers than the individuals in Group 2 did, even at a cost to them, to maintain fairness norms. In addition, the individuals in Group 1 were more concerned about the counterfactual offers from the third player. When their offers were disadvantageous compared with the counterfactual offers, the individuals in Group 1 rejected more equal or unequal offers regardless of the gain/loss context. However, the individuals in Group 2 rejected more unequal offers only in the gain context. Previous studies have found that facing unequal offers arouses negative emotions and may cause individuals to reject offers to promote egalitarianism, and this rationality could inhibit rejection behaviors to protect their benefits (Zamir, 2001; Winter and Zamir, 2005; Baumeister et al., 2007). These results are consistent with the idea that emotional decisions likely dominate Group 1 and rational decisions likely dominate Group 2. However, we found that the major similarities between the groups were that the fairness perceptions in both groups were affected not only by the responder's offer but also by the counterfactual comparison. When the counterfactual offers were more advantageous than the responder's offers, the ARs of unequal offers in both groups decreased.

### The decision processes of the log RTs and FRs affected by the counterfactual offers

Individual decision processes, such as RTs and FRs, are also modulated by counterfactual offers. According to the previous literature, in addition to ARs, RTs, and FRs are widely used indexes of decision processes in UGs (van 'tWout et al., 2005; Polezzi et al., 2008; Wright et al., 2011; Crockett et al., 2013; Gradin et al., 2015). FRs may reflect the extent to which participants perceive an offer as fair or unfair (Moretti and Di Pellegrino, 2010), whereas faster and slower RTs may imply greater ease and difficulty, respectively, in fairness-related decision-making (Wright et al., 2011). Studies have consistently revealed that ARs, RTs, and FRs are a function of the fairness of the offer (Wright et al., 2011; Crockett et al., 2013; Gradin et al., 2015). Our results revealed that with FRs and RTs as indexes, the participants reported higher FRs and made decisions more quickly when their offers were better than the counterfactual offers, even when the responders' offers were equal offers. Considering that the ARs, RTs, and FRs were related to one another in the present study, we can infer that the counterfactual offers affect fairness perceptions, even of equal offers.

### The effects of counterfactual comparisons are robust across different contexts

Similar to the results from multiple responders, in our research, we found that individuals' decision-making with multiple proposers was affected by counterfactual comparisons. People were more likely to reject an unequal offer if it left them with a more inequitable payoff relative to the other proposer's payoff, and they demanded less when they knew that the other proposer offered less. Consistent with our research, studies have found that compared with offers that are interleaved with low offers from other proposers, offers that are interleaved with higher offers from different proposers are perceived as unfair, and the rejection rates of objectively identical offers increase (Wright et al., 2011). This finding indicates that compared with advantageous offers from other proposers, a disadvantageous offer from the current proposer may be a negative outcome that increases the aversion to and rejection of unfairness (Fehr and Schmidt, 1999).

In addition to the different contexts of multiple responders or proposers, counterfactual effects are robust to the gain/loss context. Previous two-person UG studies have shown lower FRs and higher rejection rates for unequal offers in the loss context than for unequal offers in the gain context (Buchan et al., 2005; Leliveld et al., 2009; Zhou and Wu, 2011; Guo et al., 2013; Wu et al., 2014). One possible reason for this finding is that individuals are inclined to associate loss with “unfairness,” which leads unequal offers to be perceived as more unfair in the loss context than in the gain context. This association also leads offers that have higher perceived unfairness to be rejected in the loss context (Zhou and Wu, 2011). However, in contrast to a two-person UG, in our three-person UG, more acceptances overall occurred in the loss domain than in the gain domain despite the lower FRs in a three-person UG. One possible explanation is that in a three-person UG, responders might want to obtain relatively high payoffs compared with the third player's outcome. The responders could not obtain alternative offers from the proposers because the divisions were made in advance. To increase their payoffs, accepting the unequal offer might be a way to reduce the differences between the responder and the third player (Bolton and Ockenfels, 2000; Bohnet and Zeckhauser, 2004).

## Limitations

There are several limitations associated with the present study. First, although the participants were instructed that all offers were from real proposers, the current study employed hypothetical decision problems. This limitation was based on the difficulty of inviting hundreds of participants to simultaneously take part in the study. In addition, we could not ensure that every condition that we needed would appear if real participants were used. Moreover, the use of pseudo-subject situations has been widely adopted in UG studies, especially studies with multi-trials (Zhou and Wu, 2011; Alexopoulos et al., 2013; Wu et al., 2014). The findings of these UG studies suggest that fairness perceptions can be produced and studied using this approach. Thus, we used this method in our experimental design.

Second, the social comparison between the self and a third player may have contributed to our findings. Social comparison usually refers to self-other comparisons or other-other comparisons (Sandbu, 2007; Brandts and Solà, 2010; Nicklin et al., 2011; Alexopoulos et al., 2013; McDonald et al., 2013). However, counterfactual comparisons involve comparisons between the current outcome and a hypothetical outcome. Despite these differences, there are some relatively fundamental similarities between the two processes. For example, both processes are comparative judgments, can occur automatically or in a controlled fashion, have significant affective consequences, and fulfill important psychological functions of self-assessment and self-enhancement (Suls and Wheeler, 2013). These similarities reflect the profound overlap between the two processes. Therefore, excluding the potential effects of social comparisons from the counterfactual comparison may be a direction for future research to explore the specific counterfactual comparison mechanism in a multiplayer UG game.

## Conclusion

The results of this study demonstrate that regardless of the gain or loss context and the use of multiple proposers or responders, the equal or unequal offers that are given by proposers are not the only determinants of responders' judgments of fairness in a UG. The counterfactual comparisons between two different offers in similar circumstances also play an important role in responders' fairness-related decision-making processes. Fairness perceptions seem to inherently involve the fairness of social distribution and the fairness of the counterfactual comparison, which could explain the wide-ranging social debates on this issue. Specifically, such counterfactual comparisons might help explain the importance of comparative groups in salary negotiations (Babcock et al., 1996), the efficiency wage for employees in the labor market (Akerlof and Yellen, 1988), and the resolution of social conflict (Messick, 1995).

## Author contributions

Conceived and designed experiment: QL, YZ, and XL. Data collection: CW and ZY. Data Analysis: QL. Wrote the paper: QL and JT.

## Funding

This research was supported by the National Natural Science Foundation of China (Grants 31571161, 31200782, 31500872, and 31640039), the China Scholarship Council, and the National Social Science Foundation of China (Grants 14ZDB161).

### Conflict of interest statement

The authors declare that the research was conducted in the absence of any commercial or financial relationships that could be construed as a potential conflict of interest.
