# Supplementary material for: The Influence of Counterfactual Comparison on Fairness in Gain-Loss Contexts
Source: Front Psychol. 2017 May 9;8:683. doi: 10.3389/fpsyg.2017.00683 (PMC5422536; doi:10.3389/fpsyg.2017.00683)
Supplement: Supplementary file 1 [file Presentation1.PDF]

Supplementary Material

The Influence of Counterfactual Comparison on Fairness in Gain-Loss Contexts

Qi Li, Chunsheng Wang, Jamie Taxer, Zhong Yang, Ya Zheng* and Xun Liu*

*** Correspondence:** Xun Liu, Ph.D, Key Laboratory of Behavioral Science, Institute of Psychology, Chinese Academy of Sciences, 16 Lincui Road, Chaoyang District, Beijing 100101, China. Ya Zheng, Ph.D, Department of Psychology, Dalian Medical University No. 9 West Section, Lvshun South Road Dalian 116044, China. Email: liux@psych.ac.cn; zhengya@dmu.edu.cn

1 Sample participant self-introductions:

我来自农村，是一个性格开朗、活波向上、爱好运动的男生。我最喜爱运动是篮球。课余时间我喜欢读小说。我与同学和室友相处愉快，并特别享受和大家在一起的时光。我对待工作认真负责，兢兢业业。(I am an outgoing, optimistic-minded countryside boy and very interested in sports, especially in basketball. In my spare time, I like reading as well. I have built good friendships with classmates and roommates, for which I really enjoy the time with them. As for work, I am always willing to take responsibility and try my best.)
我从小学到高中都蛮喜欢学习，成绩也一直不错，但是当时我读的学校都不怎么样。大学不喜欢上课，我成绩也就很差了。我性格还算开朗，有好玩的事情经常傻乐，因此，总有人问我说你老笑什么。还有就是大家都说我比较好相处，但是其实我并不是能包容别人，只是不像其他人那样喜欢计较一些鸡毛蒜皮的事情。(From elementary school to high school, I enjoyed learning and scored well, although the schools were not very good. When I went to university, I lost interest in learning and performed poorly. Speaking of my personality, I think I'm outgoing to a degree, and sometimes I feel happy without any reasons: that's why people always ask why I am laughing. My friends think I'm easygoing, although I don’t think so. I just don't care small and trivial things as much as other people do.)
我来自吉林，比较宅，平时没事时大多都呆在宿舍。我喜欢打电子游戏 ，特别喜欢英雄联盟，不仅喜欢玩还喜欢看比赛。我爱好比较广泛，喜欢读书，听音乐，看电影电视，喜欢摄影，喜欢运动。我偶尔会出去健身。(I come from Jilin Province, in the north of China. I prefer staying at home. I am fond of playing video games, especially LoL. I am also keen on watching matches. I have many hobbies, such as reading, listening to music, watching TV, photography and sports. I occasionally go to the fitness center.)
我性格比较开朗，大方乐观，喜欢交朋友，平时也非常乐意帮助别人。大多时候乐于处在热闹的环境中，但有些时候也喜欢一个人静

静地思考。关于学习，我喜欢尝试新鲜事物，喜欢进步，所以会经常通过各种途径学习。(I'm outgoing and optimistic and like making friends and helping others. I mostly like staying in popular scenes but sometimes in a quiet place for thinking. I am up for trying new things to improve myself, so I usually study things in different ways.)
我喜欢看篮球比赛，很少去打篮球。我生活比较有规律，每天最经常的运动就是骑行。我决定要做的事会一步一步的去做，比较在意结果。我平时经常看书，自我感觉比较犟。(I like watching basketball games; however, I seldom play it. My life is a regular routine, and my daily regimen includes riding a bike. I will do what I decide to do gradually and care about the results. I often read books. I think I am a stubborn boy.)
我不太擅长和陌生人相处，但是一旦熟悉特别话唠，各种放的开。学习方面我还算努力，但是自制力不太行。(I'm not very good at getting along with strangers. I am more open and chatter nonstop if we become familiar. I think I'm hard-working in my studies; however, I don't have strong self-control.)
我是一个喜欢运动的学生。在这些运动项目中，篮球，羽毛球是我的最爱。在闲暇时间我喜欢与朋友一起出去游玩。我脾气有点大，可能是不够大度，但对人很真诚！(I am a student and like sports. Of these sports, basketball and badminton are my favorite. In my spare time, I like to go hiking with my friends. I am hot tempered because I am not generous enough. However, I am very sincere with people.)
在学习上，我对于文学类的科目比较感兴趣，计算机和线性代数曾经挂过科，都重修过了。在性格上，我有点内向，优柔寡断，最不喜欢恭迎他人。最不能忍气吞声，不会察言观色。(I'm interested in liberal arts. I failed computer science and linear algebra and restudied these two subjects. I am introverted and hesitant. What I least like is obsequious behavior. I cannot eat humble pie.)
我喜欢打篮球，每天都会适当地运动。有独立的想法和创意。我擅长沟通交流，与朋友相处打交道都挺融洽，敢于挑战困难和尝试做有意义事情。(I like playing basketball and do moderate exercise every day. I have independent and original ideas. I am good at communication and get along well with my friends. I have the courage to face difficulties and try to do meaningful things.)
我的性格内外兼有但偏内向，喜欢看电视剧，尤其是古装剧和科幻剧。喜欢思考一些哲学问题，自由不受拘束，做事大多评个人喜好。生活中爱睡觉，属于比较懒的那种，但有喜欢的事情会很积极努力的完成，喜欢挑战、爱冒险。(I am introverted. I like to watch TV, especially costume dramas and science fiction. I like to think philosophical questions and like unfettered freedom. I am a sleepy and lazy person. Nonetheless, if I like something, I will try my best to do it. Finally, I like challenge and adventure.)

2 Supplementary Analysis for Participants' Behavioral Change Over Time

We divided the trials into four segments to analyze participants' behavioral changed over time. We conducted a series of 2 (responder's offer: equal vs. unequal) × 2 (counterfactual offer: advantageous vs. disadvantageous) × 2 (context: gain vs. loss) × 4 (time segment: T1 vs. T2 vs. T3 vs. T4) repeated-measures ANOVAs to test for differences in acceptance rates and response times. For RTs, we found that there was a decrease in RTs as time increased, however, the differences of RTs in various conditions still existed, $F(3, 171) = 90.41$, $p < .001$, $\eta_p^2 = .621$ (Experiment 1) and $F(3, 198) = 82.80$, $p < .001$, $\eta_p^2 = .56$ (Experiment 2). This dictates that the decrease of RTs across time might be caused by practice. The graph of full response time distribution is presented in Figure 6 below. For acceptance rates, we found that acceptance rates in the first time segment (Mt1=0.83) were lower than in the last three time segments (Mt2=0.89, Mt3=0.88, Mt4=0.90) when the responder's loss offer was unequal in Experiment 1(see Figure 7 below). No other significant differences were found for acceptance rates. Furthermore, a regression analysis was conducted to examine the change in acceptance rates over time (First trial vs. Last trial). We did not find any significant results. These results indicated that changes of acceptance rates over time were not so noticeable in the current study.

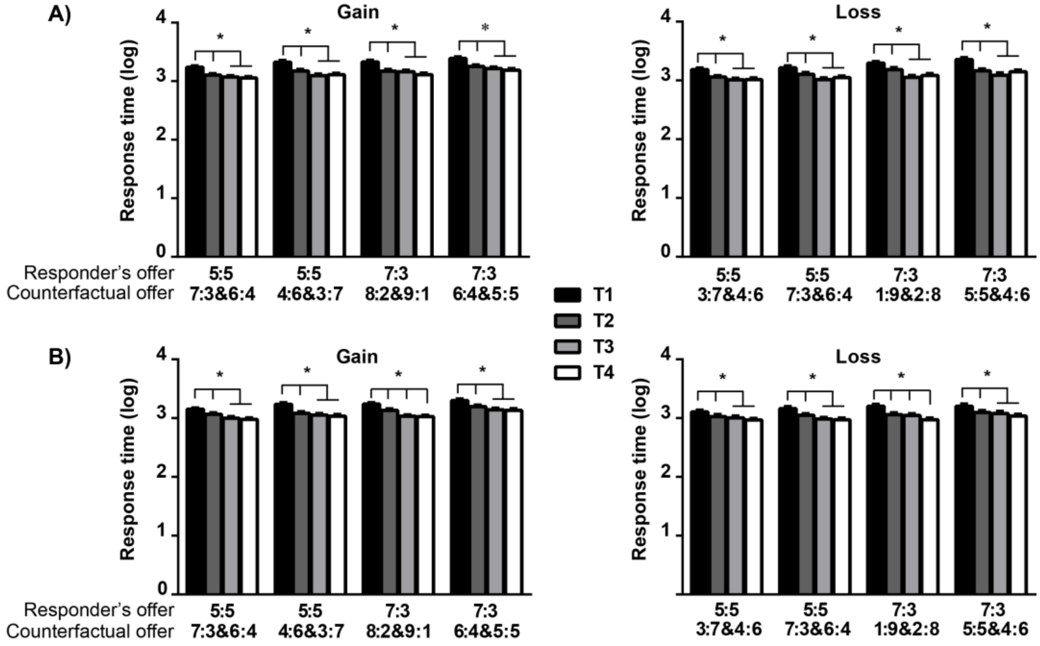

Figure 6. The log RTs of (A) Experiment 1 and (B) Experiment 2 in different time segments.

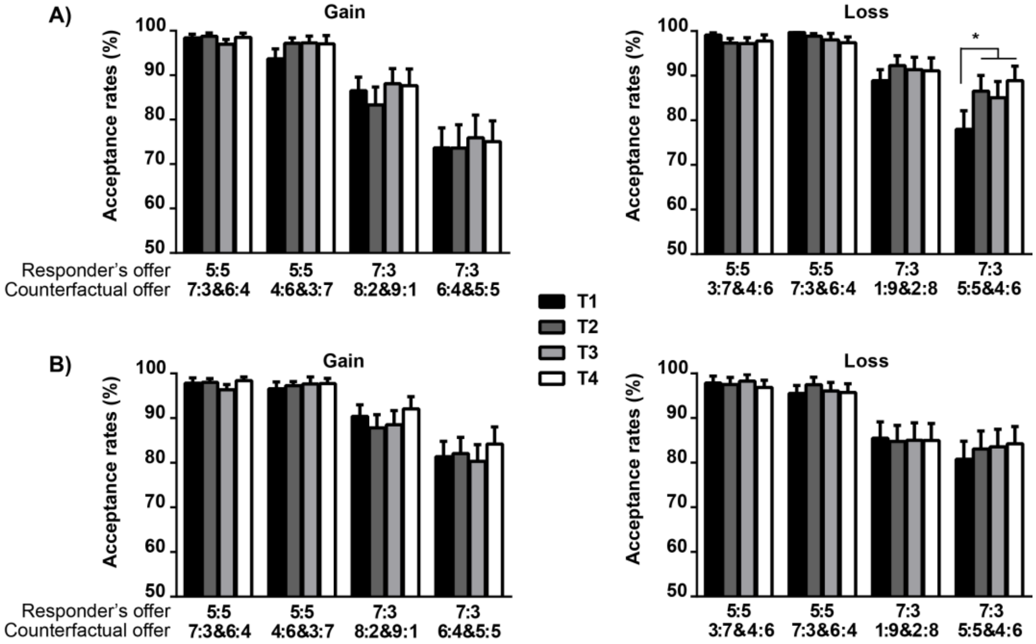

Figure 7. The acceptance rates of (A) Experiment 1 and (B) Experiment 2 in different time sections.
